# Spontaneous variability predicts compensative motor response in vocal pitch control

**DOI:** 10.1038/s41598-022-22453-0

**Published:** 2022-10-22

**Authors:** Ryosuke O. Tachibana, Mingdi Xu, Ryu-ichiro Hashimoto, Fumitaka Homae, Kazuo Okanoya

**Affiliations:** 1 Center for Evolutionary Cognitive Sciences, The University of Tokyo, Tokyo, Japan; 2 Department of Life Sciences, Graduate School of Arts and Sciences, The University of Tokyo, Tokyo, Japan; 3 Global Research Institute, Keio University, Tokyo, Japan; 4 Department of Language Sciences, Graduate School of Humanities, Tokyo Metropolitan University, Tokyo, Japan; 5 Research Center for Language, Brain and Genetics, Tokyo Metropolitan University, Tokyo, Japan; 6 RIKEN Center for Brain Science, Saitama, Japan; 7 Advanced Comprehensive Research Organization, Teikyo University, Tokyo, Japan

**Keywords:** Auditory system, Motor control, Sensorimotor processing, Human behaviour

## Abstract

Our motor system uses sensory feedback to keep desired performance. From this view, motor fluctuation is not simply ‘noise’ inevitably caused in the nervous system but would play a role in generating variations to explore better outcomes via sensory feedback. Vocalization system offers a good model for studying such sensory-motor interactions since we regulate vocalization by hearing our own voice. This behavior is typically observed as compensatory responses in vocalized pitch, or fundamental frequency (*f*_o_), when artificial *f*_o_ shifts were induced in the auditory feedback. However, the relationship between adaptive regulation and motor exploration in vocalization has remained unclear. Here we investigated behavioral variability in spontaneous vocal *f*_o_ and compensatory responses against *f*_o_ shifts in the feedback, and demonstrated that larger spontaneous fluctuation correlates with greater compensation in vocal *f*_o_. This correlation was found in slow components (≤ 5 Hz) of the spontaneous fluctuation but not in fast components (between 6 and 30 Hz), and the slow one was amplified during the compensatory responses. Furthermore, the compensatory ratio was reduced when large *f*_o_ shifts were applied to the auditory feedback, as if reflecting the range of motor exploration. All these findings consistently suggest the functional role of motor variability in the exploration of better vocal outcomes.

## Introduction

Precise control of vocal pitch, or fundamental frequency (*f*_o_), is essential for human communication since the vocal *f*_o_ is a dominant cue for prosodies in speaking or melodies in singing. A key aspect of vocal control is hearing one’s own voice, or the auditory feedback. Talkers regulate their own vocal *f*_o_ by canceling out subtle *f*_o_ deviations induced in the auditory feedback^1–4^. For example, shifting up vocal *f*_o_ in the auditory feedback elicits a response shifting down *f*_o_ in the vocalization. Such compensatory vocal response does not always cancel out the shift completely, but rather remains around half or less of the induced shift with large individual differences^5–8^. Investigating mechanisms underlying the compensatory responses for vocal *f*_o_ regulation provides opportunities to understand the adaptive audio-vocal system, which plays a critical role in our vocal control.

Recent studies in animal vocalizations, particularly in birdsongs, have suggested that variability in vocal features contributes to vocal adjustment against errors induced in the auditory feedback^9–13^. Adult songbirds typically vocalize stereotypic songs that contain almost identical acoustical patterns across renditions while exhibiting slight but unignorable variations in their acoustical features, such as *f*_o_. These variations have been reported to contribute to maintaining the song quality^13–15^. In particular, the *f*_o_ shifts in the auditory feedback elicit compensative responses of vocal *f*_o_ in birds’ song syllables^16^. The amount of this compensation becomes larger when distributions of original and shifted *f*_o_ variations are more overlapped^9,12^, linking the wider variability with the greater vocal adaptations. It has also been shown that temporal patterns of *f*_o_ fluctuation within a brief sound element have a role in keeping and improving the song quality^15,17^. Intriguingly, the vocal variability in birdsongs is not simply due to the intrinsic noise in the peripheral motor system, but a certain amount of them is actively generated by a dedicated circuit that is necessary for song learning^17–20^. These findings in songbirds’ vocalization have supported the idea that motor variations contribute to adaptive controls by generating motor exploration^11,21,22^. Such mechanisms for songbirds’ vocal control could be shared with humans^23^, especially when considering the behavioral and neural parallels between these two species for vocalization development^24–28^.

In contrast, relationships between variability and adaptability in human vocal control have not been well understood. Variability in the human vocal *f*_o_ appears to consist of several components reflecting different sources or mechanisms. These components have been classified according to their dominant frequencies in the modulation spectrum, an amplitude spectrum of *f*_o_ changing frequency (modulation frequency). For example, a quasi-periodic *f*_o_ fluctuation during singing (or *vibrato*) has been reported to show a peak around 4–7 Hz on the modulation spectrum, with greater stability in trained singers^29–31^. In contrast, non-periodic components at relatively higher modulation frequencies at 10–20 Hz, or fine fluctuation^32–34^, have been reported to be involved in the perception of voice quality both in speaking^32^ and singing^33^. Such aperiodic fast fluctuation is likely due to the physiological instability of peripheral vocal organs^35^, and hence, is less or not controllable for the central nervous system. These reports lead to a question of whether and to what extent these different components of variability could contribute to vocal regulation.

Here, we assessed associations between vocal compensatory responses against auditory feedback modifications and spontaneous variabilities of different components in vocal *f*_o_ trajectories. We tested the idea that the spontaneous variation of motor output plays a role in widening the range of exploration to pursue better performance (i.e., the motor exploration hypothesis). This hypothesis predicts that people who exhibit larger spontaneous variability in vocal *f*_o_ will show greater compensation against *f*_o_ shifts induced in the auditory feedback. In our experiment, the vocal *f*_o_ in the auditory feedback was modified while the participant was vocalizing, and the ratio of compensation in the vocalized *f*_o_ was measured for each participant. We quantified individual vocal variability that was spontaneously generated in vocalizations with unmodified feedback after separating the variability components into different modulation frequency bands. Correlation analyses between the variability and the compensation ratio across participants revealed a greater correlation for slowly fluctuating components than fast fluctuations that are likely to be less controllable in the central nervous system. Further analysis showed that the compensatory response shares the same frequency range with that of the slow component in the spontaneous fluctuation. These results are consistent with our hypothesis that spontaneous variability subserves motor explorations to enhance compensatory response against perturbations in the auditory feedback.

## Results

### Variety of the compensation ratio across participants

**Figure 1:**
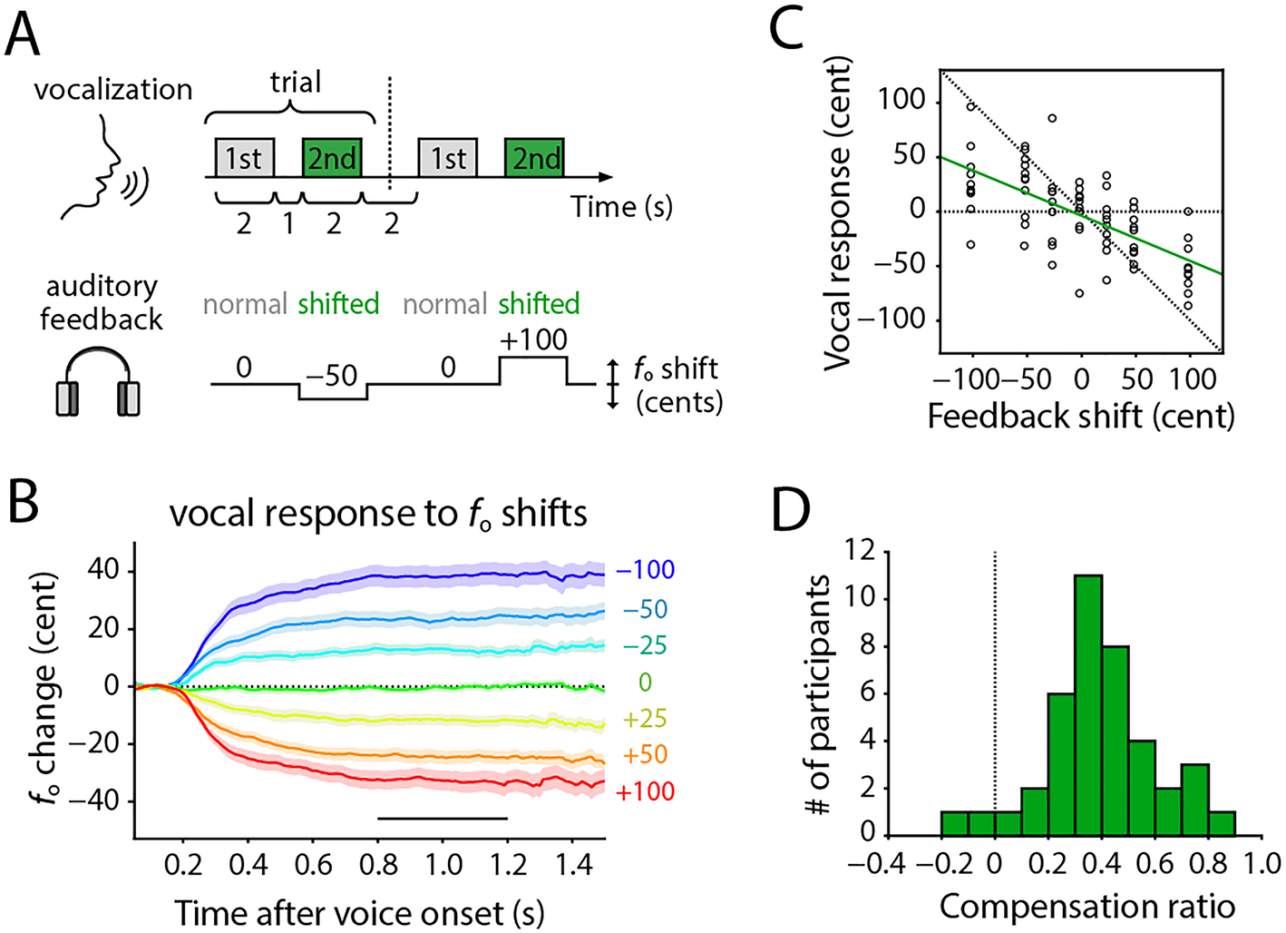
Measuring compensatory responses in vocal fundamental frequency (*f*_o_) against artificially induced *f*_o_ shifts in auditory feedback. (**A**) Schematic drawing of the experimental design. Participants vocalized twice in one trial with normal auditory feedback for the first vocalization, and with modified auditory feedback for the second. (**B**) Average of vocal *f*_o_ change across all participants in response to the seven conditions with the *f*_o_ shifts in the auditory feedback (0, ± 25, ± 50, or ± 100 cents). All trajectories were aligned at vocal onsets, and detrended before averaging (see “Methods” for detail). Pale-colored area indicates the standard error (*n* = 40). (**C**) Example of compensation amounts as a function of *f*_o_ shifts obtained from one sample participant (ID: M04). Each dot indicates the compensation amount in each trial, which was calculated as an average of the plateau period (0.8–1.2 s after voice onset) indicated as a black bar in panel (**B**). The compensation ratio was estimated as a sign-inverted value of the slope of fitted line, shown as a green line. Diagonal dotted line indicates sign-inverted unity slope. (**D**) Histogram of compensation ratios obtained from all participants. Note that here we mainly analyzed data of /a/-vocalized trials (see Fig. 6 for vowel comparisons).

In the experiment, participants were asked to continuously produce isolated vowels for 2 s twice while listening to auditory feedback via headphones, and only the second voice was modified in its feedback (Fig. 1A; see “Methods” for detail). We found a clear tendency of compensation (cancelling out) for /a/ trials in vocalized *f*_o_ against the artificially induced *f*_o_ shifts in auditory feedback (Fig. 1B). The amount of compensation was almost proportional to the amount of seven *f*_o_ shifts (0, ± 25, ± 50, or ± 100 cents), as already shown in our previous study^36^. We calculated the compensation ratio for each participant, which was defined as a sign-inverted slope of a fitted line to compensation amounts as a function of introduced *f*_o_ shifts (Fig. 1C). The obtained compensation ratio varied across participants ranging from − 0.13 to 0.82 (0.39 ± 0.21 [mean ± SD]; Fig. 1D). Note that we described results obtained from /a/-vocalize trials at first, and then assessed their generalities with /u/ trials later (see “Influence of perception and other factors” subsection).

### Variability in slow component of spontaneous fluctuations correlated with the compensation ratio

**Figure 2:**
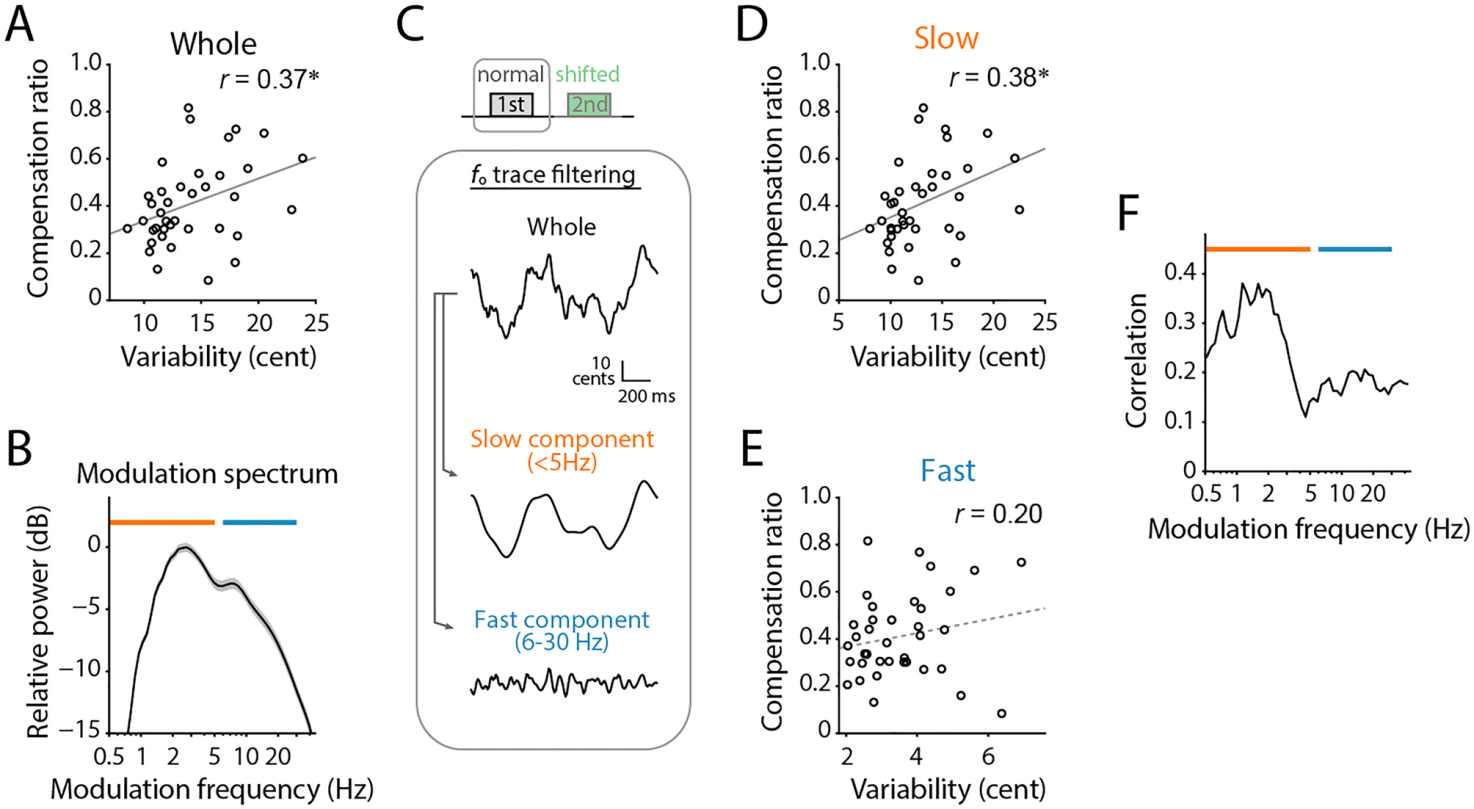
Spontaneous *f*_o_ variability during vocalizations without modification in auditory feedback, and its relationship with the compensation ratio. (**A**) The relationship between the compensation ratio and variability calculated from original (whole) *f*_o_ trajectories during no *f*_o_ shifts. Each circle indicates the data from one participant. *r* indicates Pearson’s correlation coefficient. Two participants who showed negative values in the compensation ratio were excluded as outliers. Asterisk (*) indicates statistically significant correlation (*p* < 0.05). (**B**) Modulation spectrum of spontaneous fluctuation in vocal *f*_o_ trajectories computed by a 1/2-octave filter bank (see “Methods” for detail). Gray area indicates the standard error among 40 participants. Orange and blue lines indicated frequency ranges of slow and fast fluctuation components, respectively. (**C**) Examples of filtering on the original (whole) *f*_o_ trajectory to obtain the slow and fast fluctuation components (slow: ≤ 5 Hz, fast: 6–30 Hz). (**D**, **E**) Correlation between the compensation ratio and variability of slow (**D**) or fast (**E**) fluctuation components, respectively. (**F**) Correlation coefficient between the compensation ratio and the variability of each modulation sub-band as a function of the center frequency of half-octave filter bank.

To assess to what extent the motor variability is related to the adjustment, we performed correlation analyses between the compensation ratio and several types of *f*_o_ variability. Note that we only included participants who showed compensatory responses (i.e., positive value in the compensation ratio), which resulted in excluding two out of forty participants from further analysis. To quantify vocal variability that was spontaneously generated without external perturbations, we calculated the standard deviation (SD) of an original *f*_o_ trajectory of the first vocalization (no *f*_o_ shift presented) in each trial. The mean of all SDs was defined as the variability of whole frequency components (“whole”) for each participant. This variability ranged from 8.55 to 23.87 (14.19 ± 3.72) cents. We found that the whole variability significantly correlated with the compensation ratio (Fig. 2A; Pearson’s correlation coefficient *r* = 0.37, sample size *n* = 38, *p* = 0.021). Then, we aimed to divide the whole variability into slow and fast fluctuating components according to the modulation spectrum of the spontaneous *f*_o_ fluctuation that was calculated by the 1/2-octave-band filter-bank method. The obtained modulation spectrum (Fig. 2B) showed apparent two peaks at modulation frequencies of 2–3 Hz and 6–10 Hz, suggesting two different variability components. None of the participants exhibited a sharp peak around 4–7 Hz corresponding to the presence of the vibrato component^29–31^. Thus, we defined slowly and rapidly changing components, termed as “slow” and “fast” components as having modulation frequency ranges of ≤ 5 Hz and 6–30 Hz, respectively (Fig. 2C). Obtained variabilities of slow and fast components ranged from 7.99 to 22.52 (13.07 ± 3.72) and from 2.04 to 6.93 (3.50 ± 3.72) cents, respectively.

The correlation analysis between these variabilities and the compensation ratio revealed that the slow component showed a significant correlation (Fig. 2D; *r* = 0.38, *n* = 38, *p* = 0.019), whereas the fast component did not (Fig. 2E; *r* = 0.20, *n* = 38, *p* = 0.231). Moreover, to confirm the relative impact of each modulation frequency band on the compensation, we calculated the correlation coefficients between compensation ratios and variability values in each of the subbands that were derived from the modulation spectrum analysis. This analysis showed consistent results (Fig. 2F) that the slow component (less than 4 Hz in modulation frequency) exhibited a greater correlation with the compensation ratio, but the fast one (higher than 5 Hz) did not.

### Increase of slow component in compensatory response

**Figure 3:**
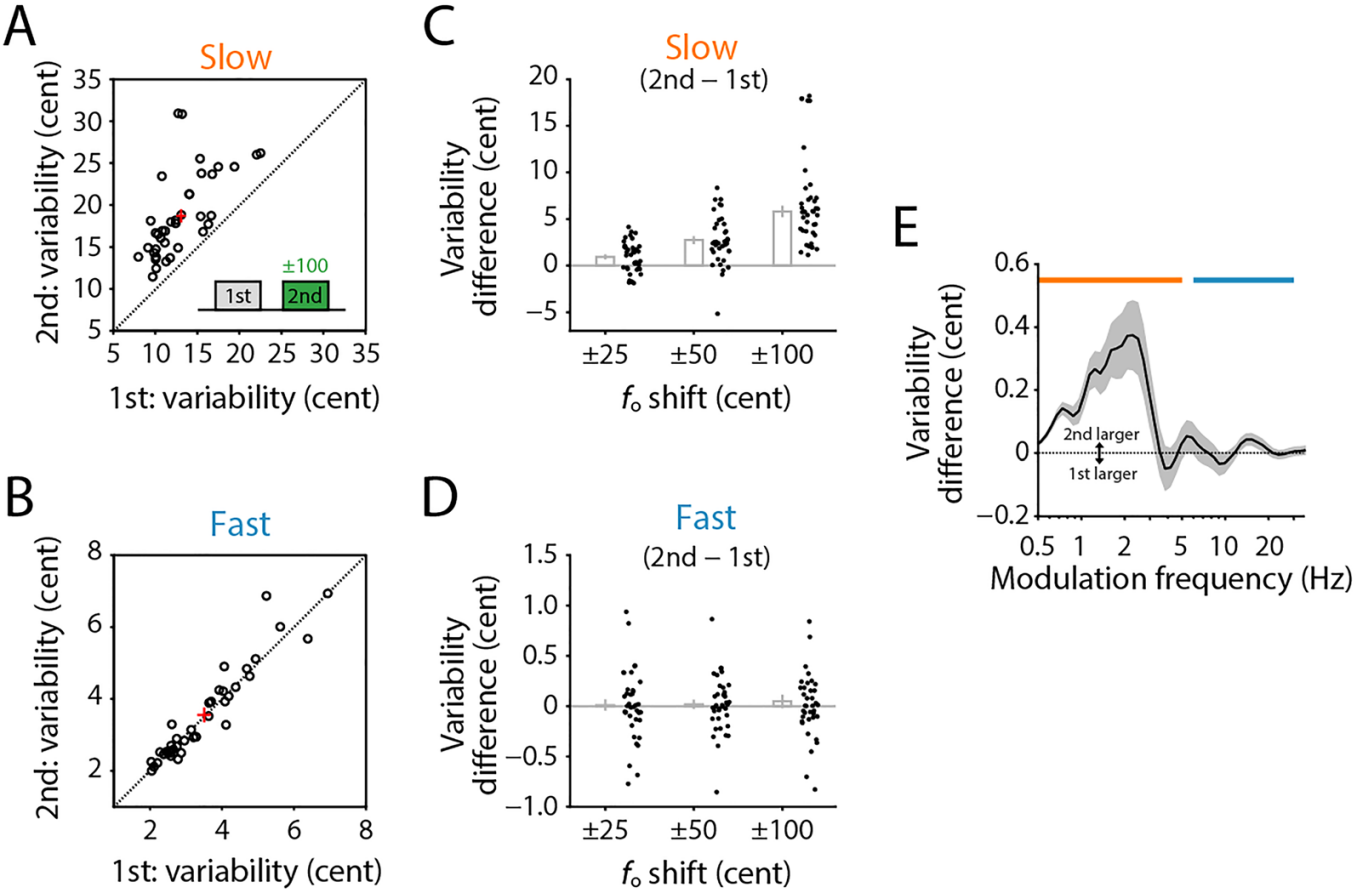
Variability comparison between the first (no *f*_o_ shifts) and second (*f*_o_ shifted) vocalization. (**A**, **B**) Mean variability of the slow (**A**), and fast fluctuation (**B**) components in ± 100-cent shift conditions of the second vocalization compared to the first vocalization. Red crosshair indicates the mean and standard error. (**C**, **D**) Variability difference of the slow (**C**) and fast (**D**) components in ± 25-, ± 50-, and ± 100-cent shift conditions between the second and first vocalizations. Errorbar indicates the standard error among 38 participants. (**E**) Variability difference of each sub-band component obtained by the modulation filter bank in ± 100-cent shift conditions between the second and the first vocalizations. Gray area indicates the standard error.

To assess which frequency component in the *f*_o_ trajectory the participants used to compensate for the *f*_o_ shifts in auditory feedback, we compared variabilities in the second vocalizations (with *f*_o_ shifts) with the first one (no shifts). We found significantly larger variability in ± 100-cent shift conditions for the slow component of the second vocalization than the first one (Fig. 3A; paired t-test, *t*(37) = 9.36, *p* < 0.001) but not for the fast component (Fig. 3B; paired t-test, *t*(37) = 0.19, *p* = 0.851). The variability difference of the second from the first vocalization increased with the increment in the *f*_o_ shift amount for the slow component (Fig. 3C) while remaining constant around zero for the fast one (Fig. 3D). These results indicated that the compensatory *f*_o_ changes contain the same ranges in modulation frequencies with the slow component of spontaneously generated vocal variability (i.e., without *f*_o_ shifts in auditory feedback). Further, we calculated the 2nd-1st variability difference in each subband derived by the modulation filter bank to confirm the modulation frequency of the compensatory *f*_o_ movement in response to auditory feedback modifications. The result (Fig. 3E) clearly depicted that the slow modulation component, which was correlated with the compensation ratio in the spontaneous fluctuation (see Fig. 2F), exhibited an extra variability in the compensatory vocal responses. This coincident finding strongly supported the idea that spontaneous variability in the slow components plays a critical role in the compensation.

### Compensation decreased with large *f*_o_ shift

**Figure 4:**
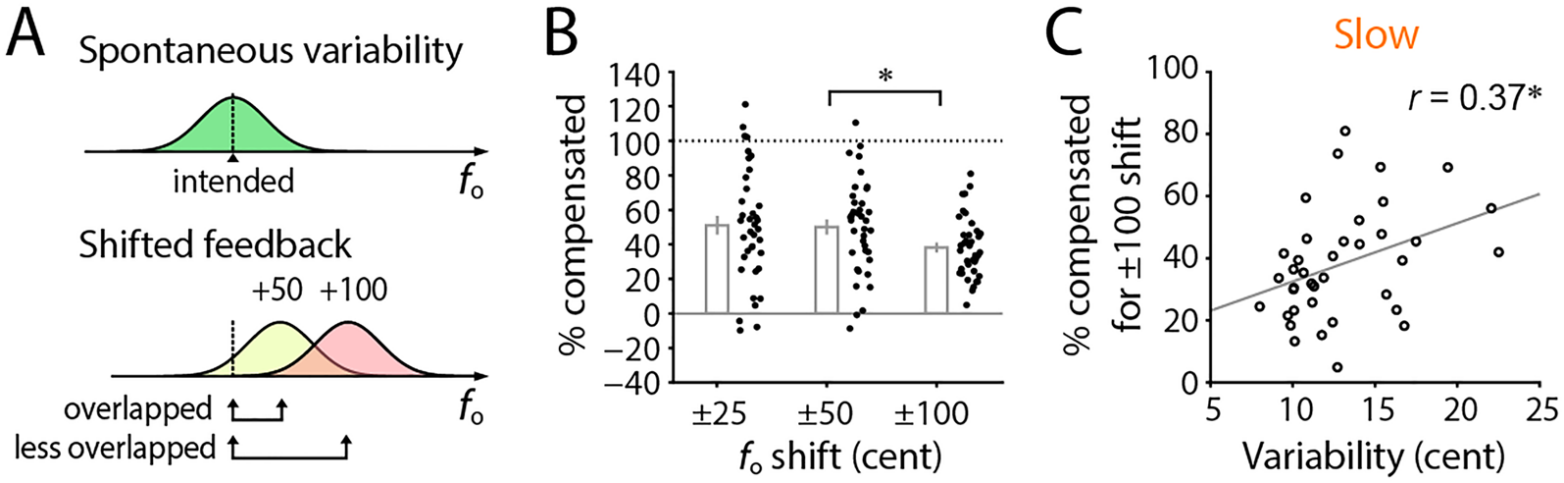
Decreased compensation for larger *f*_o_ shifts. (**A**) Schematic drawing of normalized distributions for the spontaneous *f*_o_ variability (upper) and shifted versions of its feedback after the introduction of + 50 and + 100 cent shifts (lower). Given a certain amount of variability, originally intended *f*_o_ will not overlap well with the distribution for large *f*_o_ shifts, i.e., the large *f*_o_ shifts will be outside of the motor exploration range. This can be expected to reduce the compensation ratio for that condition. (**B**) Percent amount of compensatory responses against different amounts of *f*_o_ shift (25, 50, and 100 cents). The vocal responses to positive *f*_o_ shifts were sign-inverted and averaged with that to negative shift conditions. Each dot indicates one individual participant. Error bar shows the standard error (*n* = 38). Asterisk (*) indicates statistically significant difference (*p* < 0.05 in post hoc comparisons with Tukey–Kramer correction). (**C**) Correlation between the compensation ratio for the 100-cent shift amount and the variability of the slow component. *r* shows Pearson’s correlation coefficient. Two participants who showed negative values in the compensation ratio were excluded as outliers. Asterisk (*) indicates statistically significant correlation (*p* < 0.05).

Based on the motor exploration hypothesis, we predict that the ratio of compensation to induced shift becomes small when the shift is large, as explained as follows. The spontaneous variability would work as the motor exploration. If a target *f*_o_ is within the exploration range, then the participant can find the target and adjust his/her voice toward the target. Given a certain amount of spontaneous variability, the originally intended *f*_o_ will be outside of the motor exploration range with a large *f*_o_ shift (Fig. 4A). This can reduce opportunities to find the correct (intended) *f*_o_ during vocalization, and hence, decrease the compensation ratio for such large shifts. We tested this possibility by calculating percent amounts of compensation for each of the three shift magnitudes after pooling data for positive and negative shifts by inverting its sign (Fig. 4B). Then, we found a statistically significant effect of the shift condition factor (one-way repeated ANOVA, *F*(2,74) = 3.97, *p* = 0.023). The post hoc analysis showed a significant difference between 50- and 100-cent shifts (Tukey–Kramer test; *p* = 0.002) and a marginal difference between 25- and 100-cent (*p* = 0.058), but not between 25- and 50-cent (*p* = 0.988). While the compensation in 100-cent shifts was less than in others, its correlation with the variability of the slow component was still significant (Fig. 4C; *r* = 0.37, *n* = 38, *p* = 0.022). These results consistently supported the motor exploration hypothesis in vocal control.

### Influence of perception and other factors

**Figure 5:**
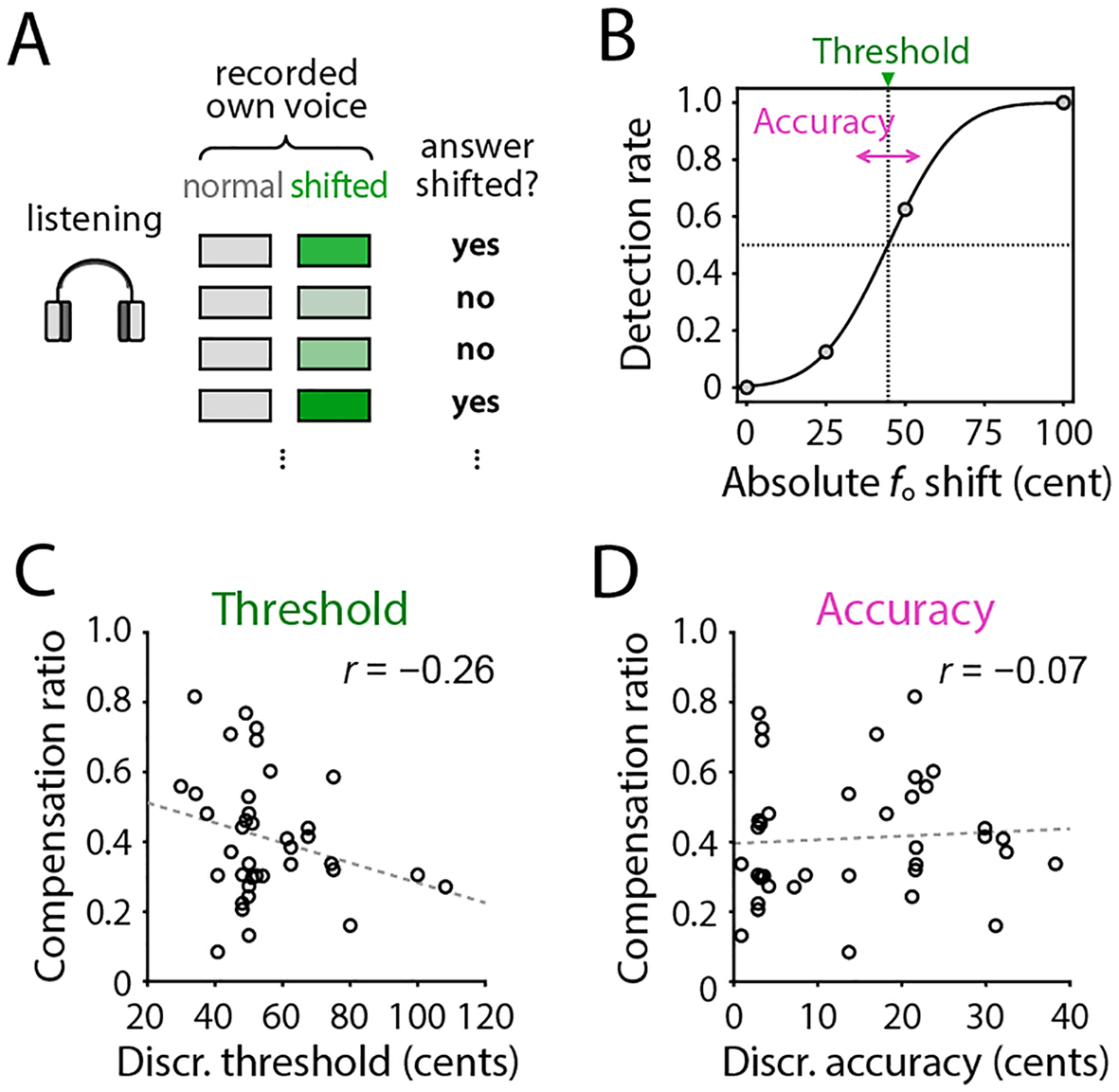
Participants’ ability to detect the *f*_o_ shifts in recorded own voices, and its correlation with the compensation ratio. (**A**) Test procedure. Participants listened to a pair of recorded voices corresponding to the first and second vocalization in each of the vocalization trials, and judged whether the second one had any modification in pitch or not. (**B**) Estimation of the detection threshold and accuracy by fitting a sigmoid function. (**C**, **D**) Correlations of the compensation ratios with the detection threshold (**C**) and accuracy (**D**). *r* shows Pearson’s correlation coefficient.

We also assessed other factors that potentially affected the compensation process, such as the perceptual ability to detect a subtle difference in vocal pitch. For this aim, we estimated participants’ ability to detect the *f*_o_ shifts induced in recorded own voices using a dataset from the listening tests performed in our previous study^36^. In this test, participants were asked to answer whether any pitch modification occurred in the second vocalization compared with the first one in each trial (Fig. 5A). We estimated the detection threshold and accuracy for noticing the presence of *f*_o_ modification by fitting a sigmoid curve on the detection rate dataset (Fig. 5B; see “Methods” for details). Obtained detection thresholds and accuracies ranged from 26.91 to 108.25 (54.71 ± 16.69) cents and from 0.87 to 38.30 (14.13 ± 11.48) cents, respectively. We then tested correlations between these perceptual properties and the compensation ratio. The result showed that the compensation ratio did not significantly correlate with either the detection threshold (Fig. 5C; *r* =  − 0.26, *n* = 38, *p* = 0.110) or accuracy (Fig. 5D; *r* = 0.07, *n* = 38, *p* = 0.694), suggesting that perceptual ability did not contribute to compensation in this case.

**Figure 6:**
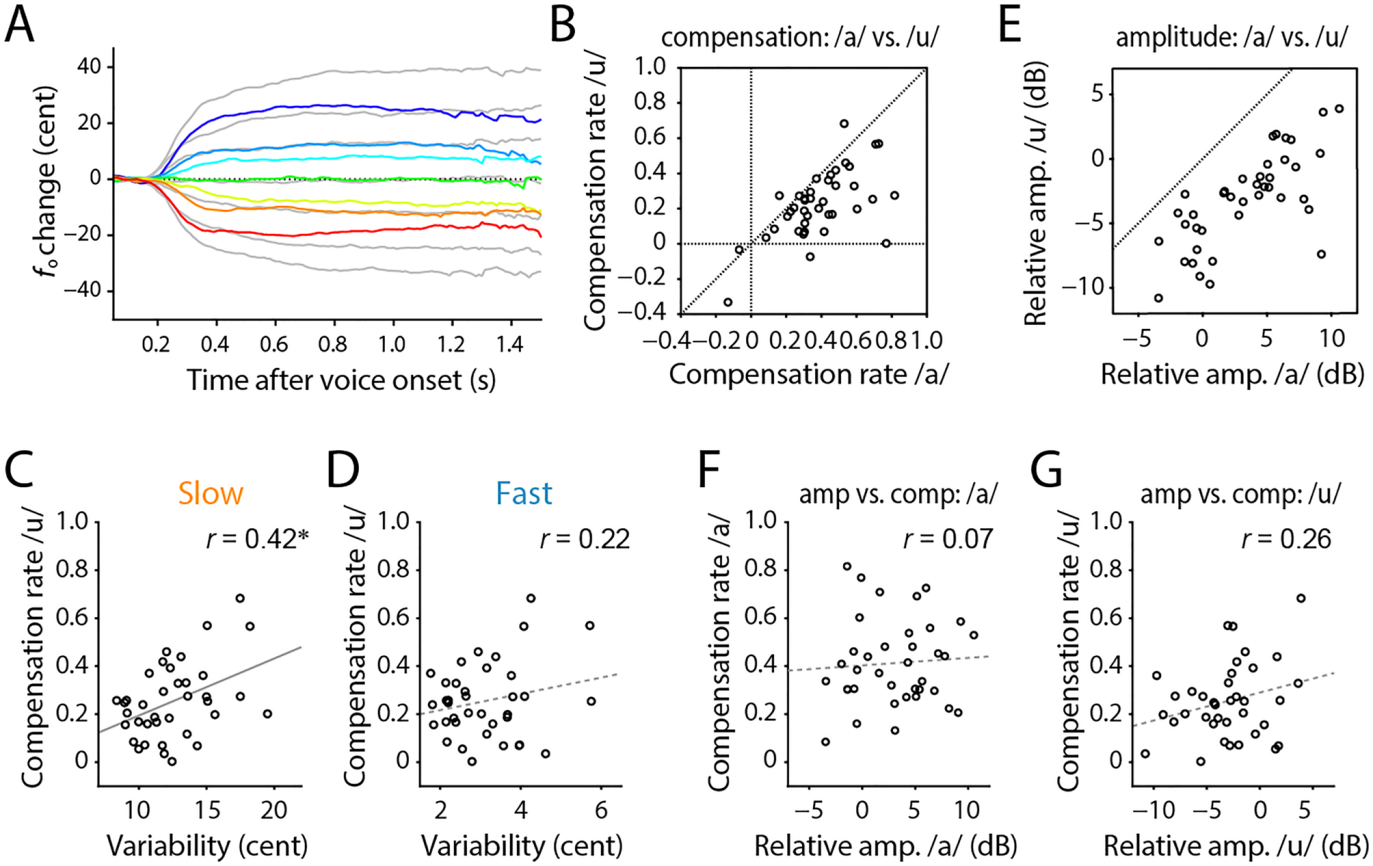
Vowel differences in compensation ratio, amplitude, and variability. (**A**) Vocal responses against *f*_o_ shifts in auditory feedback for /u/ vocalization (colored lines), those for /a/ trials as comparisons (gray lines). (**B**) The compensation ratio for /u/ vocalizations was generally smaller than that for /a/. (**C**,** D**) Correlation between the compensation ratio and variability of slow (**C**) or fast (**D**) components, respectively, for the /u/ vocalizations. (**E**) The voice amplitude of /u/ vowel was generally lower than that of /a/. (**F**,** G**) Voice amplitudes of /a/ (**F**) and /u/ (**G**) vowels did not show significant correlations with the compensation ratio. Asterisk (*) indicates significant correlation (*p* < 0.05).

Vocalizing different vowels produced different amounts of compensatory response (Fig. 6A). The compensation ratio for /u/-vocalized trials was significantly smaller than that for /a/ trials (Fig. 6B; difference: − 0.16 ± 0.18; paired t-test: *t*(39) = 5.77, *p* < 0.001). Though with a reduced degree, the compensation ratio in /u/ vocalizations exhibited a significant correlation with the spontaneous variability in their slow components (Fig. 6C; *r* = 0.42, *n* = 37, *p* = 0.010) while did not in the fast component (Fig. 6D; *r* = 0.22, *n* = 37, *p* = 0.200). Note that three out of forty participants who showed negative values in the compensation ratio for /u/ vocalizations were excluded from the correlation analysis. This consistent result among different vowels further supports the finding that the larger slow component predicts the greater compensation.

The reduced impact of the *f*_o_ shift in the /u/ vocalizations might be caused by their softer loudness of the auditory feedback than that of /a/ trials because of narrower mouth openings. The amplitude level of recorded voices was significantly lower in /u/ than in /a/ trials (Fig. 6E; difference: − 6.5 ± 2.8 dB; paired t-test: *t*(39) = 14.57, *p* < 0.001), suggesting that the relative loudness of the auditory feedback (air-conducted sound) compared to the bone-conducted feedback was lower in /u/ than in /a/ vocalization. Moreover, we tested whether the amplitude of vocalization (hence, the loudness level of auditory feedback) affected the compensation ratio. However, the relative amplitude level was not significantly correlated with the compensation ratio (Fig. 6F, G; /a/: *r* = 0.06, *n* = 38, *p* = 0.743; /u/: *r* = 0.26, *n* = 37, *p* = 0.115).

Lastly, we performed a stepwise multiple regression analysis to find the most effective model to explain the variation of the compensation ratio amongst six explanatory variables: variability in slow and fast components, detection threshold and accuracy, voice amplitude, and talker gender. The best statistical model contained only the variability in slow component as an explanatory variable (model: adjusted *R*^2^ = 0.12, *df* = 36, *SSE* = 0.168; slow component factor: *t* = 2.46, *p* = 0.019), indicating that the slow component is the main contributor for predicting the compensation ratio.

## Discussion

Recent debates on tight links between motor variability and adaptive regulation have been along with the motor exploration hypothesis, showing practical evidence in songbirds’ vocalization^9,12,13,15,16,37^, and in some other motor actions of humans^21^ or rodents^38^. Here, we provide further evidence for this debate in human vocalizations by demonstrating that the spontaneous *f*_o_ variability is positively correlated with the ratio of compensatory response against *f*_o_ shift perturbations induced in the auditory feedback (Fig. 2A). This indicates that individual participants have different intrinsic levels of motor variability, and this individual difference drives how much that person compensates for the perturbation. Our result is consistent with a previous study that used sudden *f*_o_ shifts in the auditory feedback in the middle of vocalization^8^, suggesting the robustness of this finding despite methodological differences. Further analyses showed that the slowly fluctuating components but not the fast components had a greater impact on the compensatory response (Fig. 2D, E). In addition, the compensation ratio for the largest shift conditions (± 100 cent) showed a significant decrease comparing to other shift conditions (Fig. 4C), while still exhibiting significant correlation with the spontaneous variability of slow component (Fig. 4D). These findings are consistent with the motor exploration hypothesis, which suggests that spontaneous motor variability promotes motor explorations and contributes to compensative regulation, even in vocalization processes.

Our results further indicated that the slow components of spontaneous variability contributed more to the compensation than the fast one (Fig. 2), and the main component of the compensatory response shared the same frequency range with the slow component (Fig. 3). The fast fluctuation in vocal *f*_o_ has been recognized as “microtremor” which is an involuntary fluctuation caused by physical/physiological instability^35^, suggesting that this component mainly consists of uncontrollable noise sources generated in the peripheral system. Such peripherally derived variability may not be well suited for adjustment-related motor exploration because of its uncontrollable nature^22^. In contrast, our results indicate that the slow component is controllable in the central nervous system because participants increased the amplitude of *f*_o_ fluctuation in the range of the slow component for compensatory responses. Thus, our results indicate that the slow component in spontaneous variability plays a central role in vocal compensation by generating motor exploration.

The present results fit well with the idea that variability in motor production contributes to learning by extending such exploration^21,22,39,40^, and provide further evidence supporting the generality of this hypothesis in vocal control. An alternative explanation for the variability-compensation relationship could be possible based on a factor of the perceptual ability to detect *f*_o_ changes. A previous study of vocal *f*_o_ control reported that children with less sensitive pitch discrimination abilities showed larger compensations in response to sudden induced *f*_o_ shifts^41^, suggesting a possible impact of the auditory ability on the compensation ratio. However, our results of correlation analysis between perception and compensatory response (Fig. 5) did not support this idea since they were not significantly correlated. Thus, we rule out the influence of auditory abilities but interpret the spontaneous variability as the main factor explaining the individual difference in the compensation ratio. Such dissociation between auditory perception and vocal production has been observed in a substantial population, who sing poorly in pitch but have not any problem in their hearing ability for pitch discrimination^42^.

The compensatory response data were obtained from the time window of 0.8–1.2 s after the vocal onset. Previous studies have dissociated the compensation responses into early (100–150 ms) and late (≥ 300 ms) components according to their response consistency and instruction dependency, and have associated them with “brainstem” and “cortical” pathways, respectively^3,7^. According to this dichotomy, our results obtained from the late response (0.8–1.2 s) could be associated with the cortical process. This view is consistent with findings in animal vocalization studies, which have demonstrated that interactions between the basal ganglia and cortex-homolog area play the main role in generating motor exploration and compensation for birds' song maintenance^14,43,44^.

More generally, our study suggests a shared strategy in vocal adjustment mechanisms among songbirds and humans. It should be noted that previous songbird studies have focused on variability and adjustment in a trial-by-trial manner wherein researchers assessed updating changes in vocal acoustics of every song rendition^9,10,12–14^. On the other hand, several studies have shown the importance of within-trial variability, or *f*_o_ fluctuations in one vocal element, for vocal adaptations^15,17^. Our study here measured the variability as the fluctuation in each prolonged vowel production and the adjustment as the compensatory response observed within each trial in human vocalization, while the relationship between the trial-by-trial variability and adaptive learnings over trials should be tested in future studies. Many studies have shown potential parallels in these two species in vocal learning behaviors and their neural circuitries^24,25,27,28^. Our results add further evidence of such parallels at the level of not only behavioral analogs but also computation for vocal adjustment.

## Methods

### Dataset

The dataset used in this study was originally obtained in our previous study^36^. The present study analyzed this in different ways to elucidate the relationship between the spontaneous variability and compensation behavior in vocal control. In contrast, our previous study had focused on the influences of perceptual awareness on vocal responses against various modifications to acoustical features in the auditory feedback. The different vowel data (/u/-vocalized trials) were newly analyzed in the present study. The data were obtained from forty university students (20 females; 18–26 years old) without any experience in formal music training. All the experimental procedures were approved by the Human Subjects Ethics Committee of Tokyo Metropolitan University. All participants signed informed consent forms, and all experiments were performed following relevant guidelines and regulations.

The experimental procedure was identical to that described in our previous study^36^. In brief, participants were asked to produce isolated vowels /a/ or /u/ according to the letter displayed on a computer screen while hearing auditory feedback via headphones. The auditory feedback was modified by a voice processor (Voice Worksplus, TC Helicon Vocal Technologies), and fedback to participants with masking pink noise. Participants vocalized the same vowel twice for 2 s each time with 1 s intermission in each trial, and only the second vocalization was modified in its feedback (Fig. 1A). There were 13 conditions in total for the second vocalization: 6 for pitch shifts, 6 for timbre shifts, and 1 for no shift as a control condition. In the pitch-shifted conditions, the voice spectrum was linearly expanded by ± 25, ± 50, or ± 100 cents (100 cents = one semitone), resulting in the shift of the fundamental frequency (*f*_o_). The timbre-shifted conditions expanded only the spectral envelope by ± 3, ± 6, or ± 12 percent without changing *f*_o_. There were 10 trials for each of the 13 conditions for each vowel. The order of the 260 trials was pseudo-randomized. Note that we only focused on vocal responses in the pitch-shifted conditions, but the timbre-shifted conditions were excluded from further analyses in this paper because they exhibited almost no compensative response for *f*_o_ (as reported in our previous paper^36^). We analyzed the dataset for /a/-vowel trials at first and then assessed the generality with /u/-vowel trials since the compensatory responses for /a/ trials were clearer than that for /u/ trials (see Fig. 6A, B).

After vocalization sessions, we performed a listening session (denoted as “subjective test” in our previous paper^36^) to test whether the participants noticed the sound modifications applied to their voices. We replayed participant’s voices that were recorded in two representative trials during the vocalization experiment. The participant was asked if they could perceive a change in pitch and/or timbre in the second voice compared with the first one. The present study used these responses to assess the participant’s perceptual ability to detect the presence of *f*_o_ shifts in the fedback voice.

### Preprocessing

The *f*_o_ of vocal sound was calculated by Praat 6.1^45^. The *f*_o_ calculation was performed by an adapted auto-correlation method implemented in the Praat (“To Pitch (ac)”), with 10-ms step, 40-ms window, and frequency boundaries between 75 and 600 Hz. The extracted *f*_o_ traces were converted into cent values in a logarithmic scale and obtained as follows: 1200 log_2_ (*f*_o_*/f*_*base*_), where *f*_*base*_ is a base frequency (we arbitrarily used 55 Hz for the base though this does not change the final results).

We preprocessed the obtained dataset in two steps: alignment and refinement, as described below. We firstly aligned the data by time points of vocal onsets. In this process, the vocal onset and offset were detected from the amplitude envelopes (described below) with a threshold of the background level + 30 dB. The background level was estimated from silent parts of recordings for each participant. Then, we refined the aligned data by detaching or repairing unstable/misdetected data points as follows. Fragmented data points were connected by filling brief temporal gaps (≤ 40 ms) and removing short fragments (≤ 50 ms). Unrealistic frequency jumps that were larger than ± 100 cents at the beginning part of vocalization were searched backwardly from 200-ms after the onset, and were removed. Similarly, unrealistic jumps for the ending parts were also removed by forwardly searching from 300-ms before the offset with the same threshold (± 100 cents). After these removals of unstable onset parts, we re-defined onset times as the beginning point of stable vocalization since those unstable data reflected harsh or aperiodic glottal pulsation in which participants could not sense *f*_o_ shifts in the feedback. Additionally, we also repaired the unrealistic jumps at the middle part of vocalization between 210 and 1500 ms from the vocal onset (filled with the value obtained immediately before the jump).

### Compensation ratio

To quantify compensatory responses against artificial *f*_o_ shifts in the auditory feedback, we first removed participant-specific frequency changes that were unrelated to the response to *f*_o_ shifts. For this, a common trend in all trajectories for each participant was removed by subtracting the grand mean of all trials. Moreover, we set the beginning part of each vocalization as zero by subtracting the mean value within a range of 50–150 ms in each trial to measure the responses to *f*_o_ shifts only. We defined this subtraction baseline period by visual inspection of outcomes of the grand averaging, and excluded the first 50 ms because of its instability. Then, we calculated the mean value of the late part (800–1200 ms) of data, in which the trajectories fluctuated less and were relatively stable (indicated using a black bar in Fig. 1B). We defined the compensation ratio to quantify how much the participant compensated by lowering or heightening their vocal pitch in the direction against the induced *f*_o_ shifts. This ratio was calculated as a sign-inverted slope of a line (linear regression) fitted to the mean amounts of vocal compensations as a function of *f*_o_ shifts (Fig. 1C). This measure was used to capture general tendency of the compensatory response for each participant.

### Variability assessment

To quantify the motor variability in vocalization, we calculated the standard deviation (SD) of the *f*_o_ within a period between 100 and 1200 ms after the voice onset. For this calculation, we collected *f*_o_ trajectory data of the first vocalization of each trial, in which no *f*_o_ shift was applied. We excluded data from trials that followed immediately after the *f*_o_-shifted trials to avoid contaminations of possible aftereffects. The computed SDs were averaged for each participant to obtain a variability index for the original *f*_o_ trajectories (“whole”). We extracted the slow and fast components by a low-pass filter with 5-Hz cutoff, and a band-pass filter with 6- and 30-Hz cutoff frequencies (second-order Butterworth filter), respectively. Then, we computed the mean SD of the filtered signals to obtain the variability index for a slowly fluctuating component (“slow”) or a fast fluctuating one (“fast”). These two frequency bands were defined by visual inspection of the frequency spectrum of *f*_o_ trajectories (or modulation spectrum) which is analyzed in the following subsection (Fig. 2B). Before filtering, each trajectory was zero-centered by subtracting the mean value to remove the constant component, and missing data points were filled with zero. We used the zero-phase digital filtering implemented in MATLAB software (‘filtfilt’ function).

### Modulation spectrum analysis

To assess a relative amplitude across different modulation frequencies, we calculated the modulation spectrum by a half-octave-band filter bank. We first up-sampled each *f*_o_ trajectory to a doubled rate (200 Hz), and then centered the *f*_o_ trajectory by subtracting its mean value, and filled the missing data points with zero. We defined the filter bank as a set of multiple band-pass filters that has 1/2-octave bandwidths with center frequencies equally spaced at 1/4-octave step from 0.4 to 50 Hz (second-order Butterworth filter). The amplitude of each subband was calculated as the root-mean-square value of the filtered trajectory.

### Voice amplitude calculation

The amplitude envelope of each vocalization was calculated as the root-mean-square values of an A-weighted waveform within 40-ms Hanning window for every 10-ms time step by MATLAB software. The obtained amplitude envelope was converted into a logarithmic scale (dB) by a formula: 20 log_10_ (*x*). We calculated the average value of the log-converted amplitude within a period (100–1200 ms) that includes the very beginning part of the compensatory response and the plateau part of vocalization. Then, relative values among subjects were calculated by subtracting an overall average from all participants’ data.

### Pitch-shift detection ability

We quantified the participant’s perceptual ability to detect shifts in their modified voice using the dataset obtained from the listening test performed after the vocalization sessions. We pooled trials irrespective of *f*_o_ shift directions (minus or plus), and the two vowels (/a/ and /u/) to increase the resolution and obtained 8 repetitions (2 directions × 2 vowels × 2 trials) for each absolute amount of *f*_o_ shifts. The detection rate for each absolute *f*_o_ shift was approximated by fitting a sigmoid function. For this fitting, we used a cumulative probability density function of the normal distribution as the sigmoid. Then, the detection threshold and accuracy were defined as the absolute shift value at 50% detection rate and the shallowness of fitted sigmoid (corresponding to the mean and standard deviation of the cumulative normal distribution), respectively (Fig. 5B).

### Statistical test

We tested the significance of the correlation coefficient with a significance level of *α* = 0.05. Post hoc power analysis indicated that the power (1−*β*) was 0.644 with the sample size *n* = 38 if the hypothesized correlation coefficient was *ρ* = 0.37 (the smallest number appeared in this paper with statistical significance). We also performed paired t-tests for testing differences in variability indices between the first and second vocalization (see Fig. 4), and between different vowels (see Fig. 6) at a significance level of *α* = 0.05. The repeated one-way ANOVA was performed to assess the decrease of compensation amount with the increment of *f*_o_ shifts (see Fig. 6). To examine the significance of pair-wise difference among conditions, we used the Tukey–Kramer post hoc test. Lastly, to assess the relative impact of all possible factors on the compensation ratio, we performed a stepwise multiple regression analysis. We used variability indices of slow and fast components, detection threshold and accuracy, vocal amplitude, and talker gender as regressors in the model. This analysis was performed by MATLAB program (‘stepwiselm’).

## Data Availability

Dataset and analysis scripts are available in a public repository (Open Science Framework; DOI: 10.17605/OSF.IO/CXBAU; URL: https://osf.io/cxbau/).
